# High-performance Raman quantum memory with optimal control in room temperature atoms

**DOI:** 10.1038/s41467-018-08118-5

**Published:** 2019-01-11

**Authors:** Jinxian Guo, Xiaotian Feng, Peiyu Yang, Zhifei Yu, L. Q. Chen, Chun-Hua Yuan, Weiping Zhang

**Affiliations:** 1 Quantum Institute for Light and Atoms, School of Physics and Material Science, East China Normal University, Shanghai, 200062 China; 2 School of Physics and Astronomy, and Tsung-Dao Lee Institute, Shanghai Jiao Tong University, Shanghai, 200240 China; 3 Collaborative Innovation Center of Extreme Optics, Shanxi University, Taiyuan, Shanxi 030006 China

## Abstract

Quantum memories are essential for quantum information processing. Techniques have been developed for quantum memory based on atomic ensembles. The atomic memories through optical resonance usually suffer from the narrow-band limitation. The far off-resonant Raman process is a promising candidate for atomic memories due to broad bandwidths and high speeds. However, to date, the low memory efficiency remains an unsolved bottleneck. Here, we demonstrate a high-performance atomic Raman memory in ^87^Rb vapour with the development of an optimal control technique. A memory efficiency of above 82.0% for 6 ns~20 ns optical pulses is achieved. In particular, an unconditional fidelity of up to 98.0%, significantly exceeding the no-cloning limit, is obtained with the tomography reconstruction for a single-photon level coherent input. Our work marks an important advance of atomic memory towards practical applications in quantum information processing.

## Abstract (web summary)

Storage and retrieval of memory is important for applications in quantum information processing. Here the authors demonstrate an efficient quantum Raman memory protocol by preparing hot rubidium atoms in specific states using control pulse scheme.

## Introduction

Quantum memory is a necessary component for quantum communications and quantum computing. A practical quantum memory should be efficient, low-noise, broadband, and as simple as possible to operate^1–6^. Using several approaches, including electromagnetically induced transparency (EIT), gradient echo memory (GEM), the off-resonant Faraday effect, and far off-resonant Raman memory, optical memory has been demonstrated in cold atomic ensembles^2,7–9^, atomic vapors^10–14^, and solids^15–19^. Hsiao et al.^20^ reported a 92.0% memory efficiency for a coherent light pulse in a cold atomic ensemble using EIT. Hosseini et al.^21^ used GEM to realize a 78% memory efficiency for weak coherent states with 98% fidelity. Polzik’s group^12^ demonstrated a quantum memory with a fidelity of 70% based on the off-resonant Faraday effect. These examples^12,20,21^ successfully demonstrated the capability to store optical states with high efficiency and/or fidelity exceeding the classical limit^22–24^ and sub-megahertz bandwidths. However, the bandwidth is important for the practical application of quantum memory^25^. Quantum sources with bandwidth at the GHz level have been used in long-distance quantum communication^26,27^ and quantum computers^28^.

Unlike these protocols, far-off-resonant atomic Raman memory can store short-time pulses corresponding to high bandwidths and can operate at high speeds. In addition, the far-off-resonance characteristic makes the atomic Raman memory^10,29,30^ robust against inhomogeneities in the ensemble and facilitates controlling the frequency of the output state. All of these properties indicate that atomic Raman memory has great potential in practical quantum information processing. The first experimental realization of an atomic Raman memory was demonstrated^29^ in 2010. This indeed represented significant progress in the field of Raman memory, but the limitations with low efficiency (<30%) and significant noise from the spontaneous four-wave mixing (FWM) process persist. Recently, Raman memory using photonic polarized entanglement^30^ was reported in a cold atomic ensemble with a fidelity of 86.9 ± 3.0%, but still an efficiency of only 20.9 ± 7.7%. An efficiency exceeding 50% and a fidelity exceeding 2/3 are necessary to store and retrieve an optical state within the no-cloning regime without post-selection^22–24,31^. Therefore, so far, low efficiency has appeared to exclude the broadband Raman memory as an unconditional quantum memory.

In this paper, we present an optimal control technique where the atomic vapor is performed a real-time optimal response on an input signal pulse. With a ^87^Rb atomic vapor in paraffin-coated cell at *T* = 78.5 °C, we achieve a Raman quantum memory on a coherent input of 6–20 ns duration with above 82.0% memory efficiency, and more importantly, with 98% unconditional fidelity at single photon level (*n* ≈ 1).

## Results

### Experimental setup

**Fig. 1:**
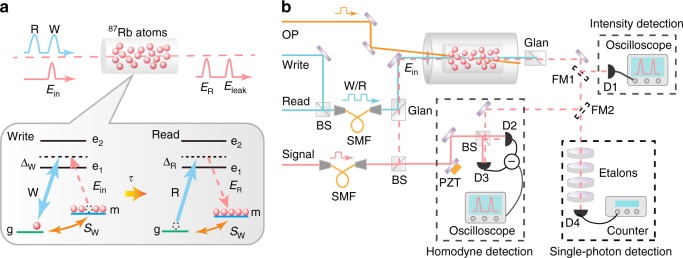
Raman memory. **a** Schematic, atomic energy levels and frequencies of the optical fields. |*g*, *m*〉: hyperfine levels |5^2^*S*_1/2_, *F* = 1, 2〉; |*e*_1_〉 and |*e*_2_〉: excited states |5^2^*P*_1/2_, *F* = 2〉 and |5^2^*P*_3/2_〉. W write field, *E*_in_ input signal, *E*_leak_ leaked signal, *S*_W_ collective atomic spin wave, R read field, *E*_R_ retrieved signal. **b** Experimental setup. The polarizations of the weak signal beams, *E*_in_ and *E*_R_, are perpendicular to the strong driving beams, W and R. The signals can be detected by homodyne detection. OP optical pumping laser, SMF single-mode fiber, BS beam splitter, PZT piezoelectric transducer. D1 photo-detector, D2 and D3 photo-diode, D4 single-photon detector, FM1 and FM2 flip mirror. The flip mirrors FM1,2 allow alternative selection of detections via intensity, homodyne, and single photon. Intensity detection is chosen to calibrate the memory efficiency by flipping FM1 up, homodyne detection combining with tomography reconstruction to determine the memory fidelity by flipping FM1 down and FM2 up, and single-photon detection to measure and analyze the excess noise in storage process by flipping FM1,2 both down

The experimental setup and atomic levels are depicted in Fig. 1. The ^87^Rb atomic vapor in the paraffin-coated glass cell is the core component of the current Raman memory. The atomic cell is 10.0 cm long, has a diameter of 1.0 cm, and is heated to 78.5 °C. Our Raman memory starts with a large ensemble of atoms that were initially prepared in the |*m*〉 = |5^2^*S*_1/2_, *F* = 2〉 state by a 44-μs-long optical pumping pulse (OP). Then, the input signal pulse *E*_in_ is stored as atomic spin excitation *S*_W_ induced by the strong off-resonant write pulse (W) with the Rabi frequency Ω_W_(*t*) and detuning Δ_W_. After a certain delay *τ*, the atomic excitation can be retrieved into optical state *E*_R_ by the strong off-resonant read pulse (R) with the Rabi frequency Ω_R_(*t*) and detuning Δ_R_. The waists of the laser beams (W, R, and *E*_in_) are all 600 μm. The two strong driving beams, W and R, can be generated by the same or different semiconductor lasers (Toptica, DLPro + Boosta) and are coupled into the same single-mode fiber. Their intensities and temporal shapes are controlled by acousto-optic modulators (AOMs). The input *E*_in_ signal comes from another semiconductor laser (Toptica, DLPro) phase-locked on the W laser. The temporal shape is controlled by a Pockels cell (Conoptics, Model No. 360-80). The shortest pulse duration of the Pockels cell is 6 ns. The W and *E*_in_ fields are two-photon resonant and spatially overlapped after passing through a Glan polarizer with 94% spatial visibility in the atomic vapor. The output signals can be separated from the strong driving pulses by another Glan polarizer with an extinction ratio of 40 dB, and detected, respectively, by intensity detection to calibrate the memory efficiency, by homodyne detection combining with tomography reconstruction to determine the memory fidelity, and by single-photon detection to analyze the excess noise in storage process. The total optical transmittance including the atomic cell and all optical elements in homodyne detection is about 89%. The four etalons with 33% transmission can filter the leaked driving photons at 115 dB.

### Efficiency

**Fig. 2:**
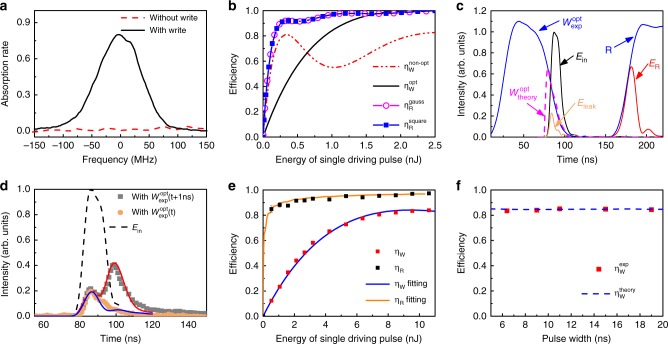
Efficient Raman memory. **a** Absorption rate of the weak input-signal pulse as a function of the Raman detuning frequency. Δ_W_ is fixed at 3.0 GHz. The input signal pulse is 10 ns long. **b** Theoretical efficiency as a function of the energy of the strong control pulse. The input optical pulse is a 10 ns near-square pulse. All optical fields detune 3.0 GHz from atomic transition and the optical depth *d* = 1100 (see Methods section for details). In the write process, the efficiency is always much smaller than 1.0 when using a non-optimal write pulse (10 ns Gaussian shape), but it can approach 1.0 with the optimal write pulse when the write pulse is larger than 1.5 nJ. In the read process, the curves with Gaussian and square read pulses coincide with each other. The retrieval efficiency is waveform-independent and increases with the energy of the read pulse until approaching 1.0. **c** Temporal modes of the strong driving (blue, experimental shape of write pulse $W_{{\mathrm{exp}}}^{{\mathrm{opt}}}$, read pulse *R*; dashed purple, theoretical shape of optimal write pulse $W_{{\mathrm{theory}}}^{{\mathrm{opt}}}$), input signal (black, *E*_in_), leaked signal (orange, *E*_leak_), and output signal (red, *E*_R_) pulses. **d** Waveform of the leaked signal with the $W_{{\mathrm{exp}}}^{{\mathrm{opt}}}(t)$ (orange circle) and $W_{{\mathrm{exp}}}^{{\mathrm{opt}}}(t + 1ns)$ (gray square) write pulse. The lines are the corresponding theoretical fits. **e** Storage efficiency (*η*_W_) and retrieval efficiency (*η*_R_) as function of the energy of the driving pulse (W and R) with the shape of $W_{{\mathrm{exp}}}^{{\mathrm{opt}}}$ and R as shown in (**c**). Square represents experimental data and solid line is theoretical fitting. The error bars correspond to one standard deviation caused by the statistical uncertainty of measurement. **f** The write-in efficiency as a function of the width of the *E*_in_ pulse

The Raman write process is a type of coherent absorption induced by a strong write pulse. As shown in Fig. 2a, when the write pulse is switched off, owing to the far-off-resonant frequency, almost 100% of the *E*_in_ pulse passes through the atomic vapor. Below, we use the total energy of such an *E*_in_ pulse to normalize the write and retrieve efficiencies. When the write pulse is turned on, part of the energy of the *E*_in_ pulse is converted coherently as the atomic spin wave *S*_W_(*z*) near the two-photon resonance frequency. The rest of the *E*_in_ energy passes through the atoms as *E*_leak_, as shown in Fig. 1a. The full width at half maximum (FWHM) of the absorption spectrum is approximately 100 MHz, as shown in Fig. 2a.

According to the theoretical analysis in ref. ^32^, the spatial-distributed atomic spin wave in a far-off-resonant Raman write process is given by

1 $$S_{\mathrm{W}}(z) = \int_0^{t_{\mathrm{W}}} q(z,t)E_{{\mathrm{in}}}(t)dt,$$

where $q(z,t) = i\frac{{\sqrt d }}{{{\mathrm{\Delta }}_{\mathrm{W}}}}{\mathrm{\Omega }}_{\mathrm{W}}^ \ast (t)e^{i\frac{{dz + h(t,t_{\mathrm{W}})}}{{{\mathrm{\Delta }}_{\mathrm{W}}}}}J_0\left( {\frac{{2\sqrt {h(t,t_{\mathrm{W}})dz} }}{{{\mathrm{\Delta }}_{\mathrm{W}}}}} \right)$, *t*_W_ is the duration of the write process, *d* is the optical depth of atomic ensemble, and $h(t,t_{\mathrm{W}}) = {\int}_t^{t_{\mathrm{W}}} \left| {{\mathrm{\Omega }}_{\mathrm{W}}(t^\prime )} \right|^2dt^\prime$ with *t*′ is the integration variable from *t t*o *t*_W_ in the co-moving frame. Eq. (1) is an iterative function that is determined by the matching between the temporal shapes of the input *E*_in_(*t*) and the write pulse Ω_W_(*t*)^32–34^. Therefore, to achieve efficient conversion, it is crucial to perform real-time control on Ω_W_(*t*) or *E*_in_(*t*) to make the atoms coherently absorb as much energy *E*_in_(*t*) as possible. The optimal control of *E*_in_(*t*) has been used to achieve efficient memory in an EIT-based process^20^, where the shape of the input signal *E*_in_(*t*) was adjusted according to atomic memory system. Here, we prefer the dynamical control Ω_W_(*t*) because a quantum memory system should have the ability to store and preserve quantum information of an input optical signal with an arbitrary pulse shape. To obtain the optimal Ω_W_(*t*), denoted $\Omega _{\mathrm{W}}^{{\mathrm{opt}}}(t)$, we first use the iterative methods mentioned in ref. ^32^ to calculate the optimal spin wave, corresponding to the minimum *E*_leak_. Then, the optimal spin wave establishes a one-to-one correspondence between *E*_in_(*t*) and $\Omega _{\mathrm{W}}^{{\mathrm{opt}}}(t)$ via Eq. (1). Thus, for any given shape of *E*_in_(*t*), $\Omega _{\mathrm{W}}^{{\mathrm{opt}}}(t)$ can be obtained from Eq. (1) via the optimal spin wave. Moreover, the corresponding optimal efficiency $\eta _{\mathrm{W}}^{{\mathrm{opt}}}$ depends only on the optical *d*epth *d* and the total energy of the write pulse. Figure 2b shows the theoretical efficiencies as the function of the energy of the strong driven pulses. Using a 10 ns near-square pulse as the input *E*_in_, the write efficiency with $\Omega _{\mathrm{W}}^{{\mathrm{opt}}}(t)$ is approximately equal to 1 when the energy of a write pulse with 10 ns duration is larger than 1.5 nJ, while the maximum write-in efficiency with a non-optimized Ω_W_(*t*) (a 10 ns Gaussian-shaped Ω_W_(*t*) is used in Fig. 2) is much smaller than one. In the read process (see Fig. 1), the spin wave *S*_W_(*z*) is retrieved back to the optical field *E*_R_(*t*) by the read pulse Ω_R_(*t*). Unlike *η*_W_, the retrieval efficiency *η*_R_ is independent of the temporal waveform^32^, and a read pulse whose duration is 10 ns with strong power but without temporal optimization is sufficient for *η*_R_ ~ 1. This can be seen in Fig. 2b. *η*_R_ increases with the total energy of the read pulse, whether Gaussian or square-shaped until *η*_R_ ~ 1. Thus, with the above optimal control on Ω_W_(*t*), the total efficiency of the Raman memory process is *η*_T_ = *η*_W_ × *η*_R_ ~ 1 in principle.

Here, we experimentally demonstrate a break of the efficiency in Raman memory with dynamic control over the temporal shape of the write pulse. In the experiment, the given *E*_in_ pulse is mapped in a forward-retrieval configuration. We derive $\Omega _{\mathrm{W}}^{{\mathrm{opt}}}(t)$ using the iteration-based optimization strategy based on the given short *E*_in_(*t*) pulse and experimentally control the temporal profile of the write pulse by using an intensity modulator (here, an AOM). The theoretical shape of optimal write pulse $W_{{\mathrm{theory}}}^{{\mathrm{opt}}}$ and the experimentally-optimized shape $W_{{\mathrm{exp}}}^{{\mathrm{opt}}}$ are given in Fig. 2c. The experimental shape is much longer than the theoretical one before the *E*_in_ pulse is turned on due to the limitation of the bandwidth of our intensity modulator, but two curves match well within the *E*_in_ duration effectively guaranteed high write-in efficiency. Furthermore, to show the definite improvement of optimization control, two write pulses are given, the optimal $W_{{\mathrm{exp}}}^{{\mathrm{opt}}}(t)$ and an optimal write pulse delayed by 1.0 ns, $W_{{\mathrm{exp}}}^{{\mathrm{opt}}}\left( {t + 1\,{\mathrm{ns}}} \right)$. The corresponding leaked optical pulses and the theoretical fits are shown in Fig. 2d. The leaked energy for the sub-optimal curve is twice that for the optimal one. Through the optimal control, the leaked energy of the input signal is greatly reduced. The storage efficiency *η*_W_, calculated by $(\overline N _{E_{{\mathrm{in}}}} - \overline N _{E_{{\mathrm{leak}}}})/\overline N _{E_{{\mathrm{in}}}}$, reaches ~84% when the atomic temperature *T* is 78.5 °C and the power of the write pulse is 10.6 nJ (Fig. 2e). Such high a write-in efficiency can be achieved when the signal duration changes from 6 to 20 ns, and *η*_W_ always remains above 83.5% by optimal control, as shown in Fig. 2f. The retrieval efficiency *η*_R_, calculated by $\left( {\overline N _{E_{\mathrm{R}}}/(\overline N _{E_{{\mathrm{in}}}} - \overline N _{E_{{\mathrm{leak}}}})} \right)$, can reach 98.5% when the read laser is 10.6 nJ, with 3.0 GHz frequency detuning (Fig. 2e). Here, *E*_in_, *E*_leak_, and *E*_R_ pulses are all measured through the intensity detection as shown in Fig. 1b. The optical paths for these pulses are arranged in the way that they are subject to the same optical losses. This allows the storage efficiency to be calibrated to characterize the atomic memory process alone. The total memory efficiency, *η*_T_ = *η*_W_ × *η*_R_, is above 82.0% when the input signal pulse contains an average number of photons ranging from 0.4 to 10^4^; thus, this Raman memory is a good linear absorber. The 82.0% memory efficiency is the best performance reported to date for Raman-based memory and far exceeds the no-cloning limit.

In principle, *η*_T_ = *η*_W_ × *η*_R_ ~ 1. With *η*_R_ = 98.5%, further improvement of *η*_T_ mainly depends on *η*_W_, which could be improved by better experimental conditions. According to our theoretical analysis, larger atomic optical depth through increasing the atomic temperature or lengthening the cell could lead to about 3% improvement. Better temporal-mode control on the W pulse may bring about 6% increase. Improving the spatial-mode match between the *E*_in_ and W beams can contribute about 5%.

### Fidelity

Fidelity is the ultimate performance criterion for quantum memory and reflects the maintenance of the quantum characteristics of the optical signal during the memory process. At the few-photon level, fidelity is readily degraded by excess noise and is mainly caused by the FWM process^10^ and spontaneous emission. Spontaneous noise comes from the spontaneous Raman scattering between the strong write pulse and the atoms populating the |*g*〉 = |5^2^*S*_1/2_, *F* = 1〉 state. Having fewer |*g*〉 atoms helps suppress the spontaneous excess noise. In our paraffin-coated cell, more than 98% of the atoms populate the |*m*〉 state. The spontaneous emission noise intensity is measured by determining the photon number using the single-photon detection as shown in Fig. 1b when the *E*_in_ pulse is turned off. On average, the spontaneous noise is approximately 0.02 photons per memory process at the end of the atomic cell for two strong driving pulses with a power of 10.6 nJ at a detuning frequency of 3.0 GHz. The FWM excess noise is mainly attributed to anti-Stokes (*AS*_FWM_, with same frequency of *E*_R_) and Stokes (*S*_FWM_) photons with the same intensity. We can deduce the proportion of *AS*_FWM_ in retrieved *E*_R_ pulse by measuring the intensity of *S*_FWM_ using single-photon detection. Our results show that the *AS*_FWM_ noise is less than 10% in *E*_R_. Such low excess noise effectively guarantees the fidelity of the quantum memory process.

**Fig. 3:**
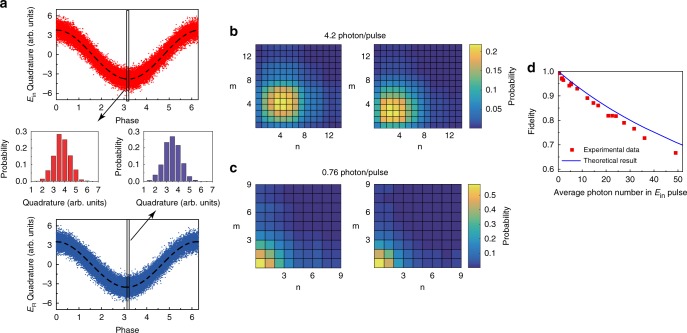
Fidelity of the Raman memory. **a** Quadrature amplitudes of the input and output signal pulses at an average of 7.9 photons/pulse. Insets are the probability distributions of the *E*_in_ and *E*_R_ quadrature values at the indicated phase. The density matrices of the input and output signal pulses at 4.2 (**b**) and 0.76 (**c**) photons/pulse on average. **d** Fidelity as a function of the number of photons contained in the input signal pulse. The red squares show the experimental data, and the black line shows the theoretical result. The error bars correspond to one standard deviation caused by the measurement noise

To achieve the fidelity performance of the current Raman quantum memory, we measure the fidelity using the equation $F = \left| {Tr\left( {\sqrt {\sqrt {\rho _{{\mathrm{in}}}} \rho _{{\mathrm{out}}}\sqrt {\rho _{{\mathrm{in}}}} } } \right)} \right|^2$^35^, where *ρ*_in_ and *ρ*_out_ are the reconstructed density matrices of *E*_in_ and *E*_R_, respectively. We record the quadrature amplitudes of the *E*_in_ and *E*_R_ signals using homodyne measurement, and we then reconstruct the density matrices by tomographic reconstruction^36^. The setup used for homodyne detection is shown in Fig. 1b. To stabilize the phase difference between the *E*_in_ and *E*_R_ pulses and simplify the homodyne setup, the write and read pulses are generated by the same laser and are controlled using one intensity modulator. In the measurement, the two weak signals, *E*_in_ and *E*_R_, are both short pulses. Matching the temporal modes of short pulses is difficult. Therefore, we use a strong continuous laser beam with the same frequency as the signal pulses *E*_in_ and *E*_R_ as the local oscillator for homodyne detection (the detailed strategy can be found in refs. ^36,37^). We recorded 10^5^ sets of quadrature amplitudes of the *E*_in_ and *E*_R_ pulses while varying the phase of the local oscillator between 0 and 2*π* by scanning the piezoelectric transducer, multiplying the quadrature amplitudes of each pulse by the temporal shapes of the corresponding signals, and finally, integrating the product over the signal pulse duration. The temporal shape functions of the *E*_in_ and *E*_R_ pulses are obtained by pointwise variance method. The integrated quadrature amplitude, which is normalized with the vacuum, as a function of the local oscillator phase is shown in Fig. 3a, where the mean number of photons contained in the *E*_in_ pulse is 7.9. The phase of the retrieved *E*_R_ signal pulse closely follows that of the input *E*_in_ pulse. Insets in Fig. 3a are the probability distributions of amplitude quadratures of the *E*_in_ and *E*_R_ pulses showing good Gaussian distributions.

The density matrix elements of the *E*_in_ and *E*_R_ pulses are obtained based on the quadrature-amplitude results using the maximum-likelihood reconstruction method^36,38^. Then on the basis of the diagonal density matrix elements, the photon distributions of the input and output pulses can be achieved to calculate the average photon numbers. The results are plotted in Fig. 3b, c, with the input pulses containing, on average, 4.2 and 0.76 photons, corresponding to unconditional fidelities of 0.915 and 0.98, respectively. The fidelities significantly exceed the no-cloning limit, indicating that the current Raman memory is a quantum-memory process and does not introduce significant excess noise during the memory process.

As mentioned above, the current Raman memory is a good linear absorber and allows the storage and retrieval of coherent optical signals at the single-photon level for up to 10^4^ photons with the same memory efficiency. Unlike the efficiency, the unconditional fidelity of the quantum memory of the coherent field is related to the average photon number contained in the input signal $\left( {\overline N _{E_{{\mathrm{in}}}}} \right)$ and efficiency (*η*_T_) by $F = 1/\left[ {1 + \overline N _{E_{{\mathrm{in}}}}(1 - \sqrt {\eta _{\mathrm{T}}} )^2} \right]$^24^, which shows that if *η*_T_ < 1, the fidelity will rapidly decrease with $\overline N _{E_{{\mathrm{in}}}}$ owing to the worse overlap between *ρ*_in_ and *ρ*_out_. In Fig. 3d, the fidelity is shown as a function of $\overline N _{E_{{\mathrm{in}}}}$ with *η*_T_ = 82.0%. The experimental *F* value is slightly smaller than the theoretical *F* value because of the excess noise in the experiment. *F* exceeds the no-cloning limit^22–24^ at $\overline N _{E_{{\mathrm{in}}}} \le 49$ in the current Raman memory process.

### Bandwidth and coherence time

Far off-resonant Raman memory is a genuine broadband memory. The ability to store and retrieve broadband pulses was successfully demonstrated in ref. ^29^, where a bandwidth larger than 1 GHz of the retrieved signal was obtained using a 300 ps and 4.8 nJ read pulse. In a practical Raman memory, the bandwidth is generated dynamically by the strong driving pulses. In Fig. 2, the shortest *E*_in_ pulse has a FWHM of 6 ns and a bandwidth of 170 MHz. The FWHM of the *E*_R_ signal pulse which mainly depends on the rise time of the R pulse is 13 ns, corresponding to a bandwidth of 77 MHz. The bandwidth in the current memory is dozens or hundreds of times larger than the values reported based on the EIT^20^, Faraday^12^, and GEM^21^ approaches, thus demonstrating the broadband memory ability of the current quantum memory scheme.

In our experiment, the memory bandwidth is limited by the currently available intensity modulators (AOM and Pockels cell) and the corresponding electronics controllers (arbitrary wave generators) in our lab. The duration limit of the Pockels cell is 6 ns. As shown in Fig. 2f, the write-in efficiencies remain above 83.5% as the signal duration is larger than 6 ns. Shorter *E*_in_ and *E*_R_ pulses corresponding larger bandwidths need faster intensity modulators and electronics controllers.

The decoherence time, which is another essential criterion for good quantum memory, is measured to be approximately 1.1 μs. The delay-bandwidth product at 50% memory efficiency, an appropriate figure of merit, is defined as the ratio of the memory time to the duration of the signal pulse and is 86 in this work. In the present atomic system, the decoherence time is mainly limited by the atomic diffusion out of the laser beam^39^. The delay-bandwidth product could be increased to ~10^3^ by using a shorter signal pulse and an anti-relaxation-coated cell with the same diameter of the millimeter order as the laser beams. This can lead to a typical decoherence time of approximately several microseconds^40^.

## Discussion

In summary, we have demonstrated a high-performance broadband quantum optical memory via pulse-optimized Raman memory in free space. The 82.0% memory efficiency is the highest value obtained to date for far-off-resonant Raman memory. The unconditional fidelity of 98% for an input pulse containing an average of approximately one photon significantly exceeds the classical limit. The 77 MHz bandwidth of the current memory is dozens or hundreds of times larger than the reported bandwidths for memories based on the EIT, Faraday, and GEM approaches. The delay-bandwidth product at 50% memory efficiency is 86. These attractive properties demonstrate that the Raman memory is a high-performance broadband quantum memory. Additionally, our memory is implemented in an atomic vapor system that can be easily operated and could become the core of a scalable platform for quantum information processing, long-distance quantum communication and quantum computation.

## Methods

### Optical depth

The formula of the optical depth is *d* = *g*^2^*NL*/(*γc*)^32^, where *g* is the atom-field coupling constant, *N* is the number of atoms, *L* is the length of atomic ensemble, *γ* is the decay rate of the |*e*_1_〉, and *c* is the speed of light. The values of these parameters are: *g* = 6.79 × 10^4^ s^−1^ and *γ* = 3.613 × 10^7^ s^−1^ for ^87^Rb D1 line, *L* = 10 cm, *c* = 3 × 10^8^ m ⋅ s^−1^. The number of atoms varies with the temperature of the vapor cell. According to fluorescence measurement in experiment^41^, *N* ~ 2.58 × 10^10^ at the cell temperature of 78.5 °C.

## Data Availability

The data that support the findings of this study are available from the corresponding authors upon reasonable request.

## References

[1] Fleischhauer M, Lukin MD (2000). Dark-state polaritons in electromagnetically induced transparency. Phys. Rev. Lett..

[2] Liu C, Dutton Z, Behroozi CH, Hau LV (2001). Observation of coherent optical information storage in an atomic medium using halted light pulses. Nature.

[3] Phillips DF, Fleischhauer A, Mair A, Walsworth RL, Lukin MD (2001). Storage of light in atomic vapor. Phys. Rev. Lett..

[4] Mair A, Hager J, Phillips DF, Walsworth RL, Lukin MD (2002). Phase coherence and control of stored photonic information. Phys. Rev. A.

[5] Honda K (2008). Storage and retrieval of a squeezed vacuum. Phys. Rev. Lett..

[6] Appel J, Figueroa E, Korystov D, Lobino M, Lvovsky AI (2008). Quantum memory for squeezed light. Phys. Rev. Lett..

[7] Zhao R (2008). Long-lived quantum memory. Nat. Phys..

[8] Bao XH (2012). Efficient and long-lived quantum memory with cold atoms inside a ring cavity. Nat. Phys..

[9] Parniak M (2017). Wavevector multiplexed atomic quantum memory via spatially-resolved single-photon detection. Nat. Commun..

[10] Reim KF (2011). Single-photon-level quantum memory at room temperature. Phys. Rev. Lett..

[11] Hosseini M, Sparkes BM, Campbell G, Lam PK, Buchler BC (2011). High efficiency coherent optical memory with warm rubidium vapour. Nat. Commun..

[12] Julsgaard B, Sherson J, Cirac JI, Fiurasek J, Polzik ES (2004). Experimental demonstration of quantum memory for light. Nature.

[13] van der Wal CH (2003). Atomic memory for correlated photon states. Science.

[14] Pu YF (2017). Experimental realization of a multiplexed quantum memory with 225 individually accessible memory cells. Nat. Commun..

[15] Longdell JJ, Fraval E, Sellars MJ, Manson NB (2005). Stopped light with storage times greater than one second using electromagnetically induced transparency in a solid. Phys. Rev. Lett..

[16] Chaneliere T, Ruggiero J, Bonarota M, Afzelius M, Le Gouët JL (2010). Efficient light storage in a crystal using an atomic frequency comb. New J. Phys..

[17] Bigelow MS, Lepeshkin NN, Boyd RW (2003). Superluminal and slow light propagation in a room-temperature solid. Science.

[18] Clausen C (2011). Quantum storage of photonic entanglement in a crystal. Nature.

[19] England DG (2015). Storage and retrieval of THz-bandwidth single photons using a room-temperature diamond quantum memory. Phys. Rev. Lett..

[20] Hsiao YF (2018). Highly efficient coherent optical memory based on electromagnetically induced transparency. Phys. Rev. Lett..

[21] Hosseini M, Campbell G, Sparkes BM, Lam PK, Buchler BC (2011). Unconditional room-temperature quantum memory. Nat. Phys..

[22] Grosshans F, Grangier P (2001). Quantum cloning and teleportation criteria for continuous quantum variables. Phys. Rev. A.

[23] Hétet G, Peng A, Johnsson MT, Hope JJ, Lam PK (2008). Characterization of electromagnetically-induced-transparency-based continuous-variable quantum memories. Phys. Rev. A.

[24] He QY, Reid MD, Giacobino E, Cviklinski J, Drummond PD (2009). Dynamical oscillator-cavity model for quantum memories. Phys. Rev. A.

[25] Simon C (2010). Quantum memories. Eur. Phys. J. D.

[26] Halder M (2008). High coherence photon pair source for quantum communication. New J. Phys..

[27] Rakher MT (2011). Simultaneous wavelength translation and amplitude modulation of single photons from a quantum dot. Phys. Rev. Lett..

[28] Lounis B, Orrit M (2005). Single-photon sources. Rep. Prog. Phys..

[29] Reim KF (2010). Towards high-speed optical quantum memories. Nat. Photonics.

[30] Ding DS (2015). Raman quantum memory of photonic polarized entanglement. Nat. Photonics.

[31] Varnava M, Browne D, Rudolph T (2006). Loss tolerance in one-way quantum computation via counterfactual error correction. Phys. Rev. Lett..

[32] Gorshkov AV, André A, Lukin MD, Sørensen AS (2007). Photon storage in Λ-type optically dense atomic media. II. Free-space model. Phys. Rev. A.

[33] Nunn J (2007). Mapping broadband single-photon wave packets into an atomic memory. Phys. Rev. A.

[34] Wasilewski W, Raymer MG (2006). Pairwise entanglement and readout of atomic-ensemble and optical wave-packet modes in traveling-wave Raman interactions. Phys. Rev. A.

[35] Nielsen MA, Chuang IL (2000). Quantum Computation and Quantum Information.

[36] Paris M, Řeháček J (2004). Quantum State Estimation.

[37] MacRae A, Brannan T, Achal R, Lvovsky AI (2012). Tomography of a high-purity narrowband photon from a transient atomic collective excitation. Phys. Rev. Lett..

[38] Lvovsky AI (2004). Iterative maximum-likelihood reconstruction in quantum homodyne tomography. J. Opt. B: Quantum Semiclass. Opt..

[39] Camacho RM, Vudyasetu PK, Howell JC (2009). Four-wave-mixing stopped light in hot atomic rubidium vapour. Nat. Photonics.

[40] Klein M (2009). Slow light in narrow paraffin-coated vapor cells. Appl. Phys. Lett..

[41] Zhao M, Zhang K, Chen LQ (2015). Determination of the atomic density of rubidium-87. Chin. Phys. B.

